# Early start of progressive motor deficits in Line 61 α-synuclein transgenic mice

**DOI:** 10.1186/s12868-017-0341-8

**Published:** 2017-01-31

**Authors:** R. Rabl, C. Breitschaedel, S. Flunkert, S. Duller, D. Amschl, J. Neddens, V. Niederkofler, E. Rockenstein, E. Masliah, H. Roemer, B. Hutter-Paier

**Affiliations:** 1 QPS Austria GmbH, Parkring 12, 8074 Grambach, Austria; 2 Institute of Zoology, Karl Franzens University, Graz, Austria; 3 Department of Pathology, University of California San Diego, La Jolla, CA USA

**Keywords:** α-Synuclein, Parkinson’s disease, Synucleinopathies, Protein expression, Motor deficits, Memory deficits

## Abstract

**Background:**

Synucleinopathies such as Parkinson’s disease or multiple system atrophy are characterized by Lewy bodies in distinct brain areas. These aggregates are mainly formed by α-synuclein inclusions, a protein crucial for synaptic functions in the healthy brain. Transgenic animal models of synucleinopathies are frequently based on over-expression of human wild type or mutated α-synuclein under the regulatory control of different promoters. A promising model is the Line 61 α-synuclein transgenic mouse that expresses the transgene under control of the Thy-1 promoter.

**Results:**

Here, we show an extended characterization of this mouse model over age. To this end, we analyzed animals for the progression of human and mouse protein expression levels in different brain areas as well as motor and memory deficits. Our results show, that Line 61 mice exhibited an age dependent increase of α-synuclein protein levels in the hippocampus but not the striatum. While murine α-synuclein was also increased with age, it was lower expressed in Line 61 mice than in non-transgenic littermates. At the age of 9 months animals exhibited increased neuroinflammation. Furthermore, we found that Line 61 mice showed severe motor deficits as early as 1 month of age as assessed by the wire hanging and nest building tests. At later ages, initial motor deficits were validated with the RotaRod, pasta gnawing and beam walk tests. At 8 months of age animals exhibited emotional memory deficits as validated with the contextual fear conditioning test.

**Conclusion:**

In summary, our results strengthen and further expand our knowledge about the Line 61 mouse model, emphasizing this mouse model as a valuable in vivo tool to test new compounds directed against synucleinopathies.

## Background

The pathology of Parkinson’s disease (PD) is characterized by dopaminergic neuron loss in the substantia nigra as well as Lewy bodies in distinct brain areas. Lewy bodies are mainly formed by α-synuclein (α-Syn) inclusions and are typical for idiopathic as well as familial forms of PD [1]. Since α-Syn aggregates are also found in other neurodegenerative diseases such as dementia with Lewy bodies and multiple system atrophy, all these pathologies are summarized as synucleinopathies. Aggregation of α-Syn can be caused by different point mutations [2, 3] or over-expression of the wild type protein due to duplication or triplication of the α-Syn gene SNCA [4–6]. In order to model synucleinopathies in vivo, different mouse models have been developed [7–10]. Here we used Line 61 mice that over-express human wildtype α-Syn under control of the Thy-1 promoter [8]. Previous experiments with Line 61 mice revealed motor deficits starting at an age of 2 months [11]. Their behavioral results match well with biochemical and histological results by Rockenstein et al. [8] whose initial characterization shows high human α-Syn expression throughout the Line 61 mouse brain, including the basal ganglia, thalamus and substantia nigra. Additionally, Chesselet and colleagues analyzed Line 61 animals for motor and cognitive deficits at the age of 4–5 months, confirming results of earlier studies [11, 12].

The present study verifies previously published data, but additionally shows human and murine α-synuclein protein expression over age, neuroinflammation in adult animals and documents first motor deficits already at an age of 1 month that progress over age. Furthermore, we were able to determine emotional learning deficits at an age of 8 months.

## Methods

### Animals

For this study male mice, overexpressing human wild-type α-Syn under the regulatory control of the Thy-1 promoter with a C57BL/6xDBA background were used (Line 61 [8]). For behavioral tests 1 to 8-month old animals were used. At least eight animals per group were analyzed. All experiments were performed with hemizygous Line 61 mice and corresponding non-transgenic littermates.

Animals were housed in individually ventilated cages on standardized rodent bedding (Rettenmayer^®^) in the AAALAC accredited animal facility of QPS Austria. The room temperature was kept at approximately 21 °C and the relative humidity between 40 and 70%. Mice were housed under constant light-cycle (12 h light/dark). Dried pelleted standard rodent chow (Altromin^®^) and normal tap water were available to the animals ad libitum. Each individual animal was checked regularly for any clinical signs.

### Behavioral tests

All behavioral tests were performed during the light phase and animals were habituated to the experimental room for at least 30 min.

#### Wire hanging test

The test is also called wire suspension test. A standard wire cage lid was used. The mouse was placed on the top of the lid. Afterwards, the lid was slightly shaken to cause the mouse to grip the wires, and then turned upside down. Duct tape placed around the perimeter of the lid prevented the mouse from climbing over the edge. The latency to fall off the wire lid was measured in seconds. A 90 s cut-off time was used.

#### RotaRod

The test assesses motor coordination by placing animals on a rotating rod (five-lane-Rota Rod; Ugo Basile, Italy) that runs at a constant or an accelerating speed. The apparatus automatically records the latency to fall as well as the speed at fall. All mice were tested on three days. After a 180 s training session at a constant speed of 2 rpm approximately 1 h before testing on day 1, each animal was exposed to the apparatus for a 180 s testing session. The initial speed increased from 2 to 20 rpm over a period of 180 s in the test sessions. On day 2 and 3, the test was performed without training [13].

#### Pasta gnawing test

This test was developed to study fine motor deficits in small rodents and adapted from the pasta handling test [14, 15]. Several 1 cm long dry spaghetti noodles were provided to each single housed animal. A microphone was placed above the cage and as soon as the animal started eating, the recording started. Recording time was approximately 1 min depending on the intensity of eating behavior. Biting was recorded using the Behringer ECM 8000 microphone with a Steinberg CL1 sound card. The number of bites per gnawing episode and the biting frequency were evaluated using Avisoft SAS Lab Pro program (Avisoft Bioacoustics, Germany).

#### Nest building

To test the individual nest building behavior, mice were single housed in cages containing wood chip bedding and one 5 × 5 cm square of pressed cotton (‘nestlet’). The following morning the nest was assessed according to a five-point scale as follows: (1) Nestlet not noticeably touched (>90% intact). (2) Nestlet partially torn up (50–90% remaining intact). (3) Mostly shredded but often not identifiable nest site: <50% of the Nestlet remains intact but <90% is within a quarter of the cage floor area, i.e. the cotton is not gathered into a nest, but spread around the cage. (4) An identifiable, but flat nest: >90% of the Nestlet is torn up, the material is gathered into a nest within a quarter of the cage floor area, but the nest is flat, with walls higher than mouse body height (curled up on its side) on less than 50% of its circumference. (5) A (near) perfect nest: >90% of the Nestlet is torn up, the nest is a crater, with walls higher than mouse body height on more than 50% of its circumference [16].

#### Beam walk test

Mice were trained and tested to traverse an elevated narrow beam, which was brightly illuminated at the start and ended in their home cage. Animals were three times trained on a square training beam that is slightly wider than the square testing beam. At the same day, a square beam of 11 mm width and a round beam of 16 mm in diameter were used for testing. The testing phase was videotaped and analyzed manually. The time to traverse the beam and the number of foot slips were analyzed.

#### Contextual fear conditioning

The fear conditioning test was conducted in an automated box (TSE-Systems, Germany). Mice were trained and tested on 2 consecutive days. On the training day, mice received a foot shock (0.5 mA, 2 s) 5 s after being placed into the conditioning chamber. 30 s afterwards mice were returned to their home cage. 24 h after training, mice were tested by being returned to the conditioning chamber for 5 min without any shock, and freezing behavior was recorded by the automated system and evaluated separately for every minute.

### Tissue preparation

Tissue was taken from 2-, 3- and 6-month old Line 61 mice and non-transgenic littermates. Mice were deeply anesthetized by Isoflurane (Baxter, Austria) and the thorax was opened to excavate the heart. Animals were flush-perfused transcardially with 0.9% saline through the left ventricle. The hemispheres were divided at midline. One hemisphere was immersion-fixed in 4% paraformaldehyde in 0.1 M phosphate buffer, pH 7.4, for 2 h at room temperature (RT), was then cryoprotected in 15% sucrose in PBS overnight, embedded in tissue freezing medium (Leica Biosystems, Germany) in cryomolds and was snap-frozen in dry ice-cooled liquid isopentane. The frozen samples were then stored at −80 °C until sectioning. The other hemisphere was dissected into hippocampus, striatum and rest brain and shock-frozen on dry ice and stored at −80 °C for α-Syn determination.

### Biochemistry

Protein was extracted in buffer containing 20 mM Tris–HCl, pH 7.4, 50 mM NaCl, 1% Triton X-100 including freshly added 0.2 mM sodium-orthovanadate protease inhibitor cocktail (Calbiochem, Germany) and phosphatase inhibitor cocktail (Sigma, USA) by using the tissue ruptor (Qiagen, Germany). Lysates were incubated for 30 min on ice, followed by centrifugation at 15,000*g* for 60 min at 4 °C. The pellet and supernatant were collected as the Triton X-100-insoluble and soluble fractions, respectively. The Triton X-100-insoluble pellets were dissolved in the previously described lysis buffer containing 2% SDS. Aliquots of the resulting fractions were stored at −80 °C until further analyses.

α-Syn levels in the Triton-X-100 soluble and insoluble fraction [17] were determined by using a commercially available immunosorbent assay (K151TGD-4; MesoScale Discovery, USA) according to the manufacturer’s protocol for human α-Syn levels. Evaluation of murine α-Syn levels was performed by a self-made MesoScale Discovery plate using the murine specific rabbit anti-murine α-synuclein mAb (clone: D37A6, # 4179, Cell Signaling, USA). α-Syn levels were evaluated in comparison to a recombinant α-synuclein protein (residues 1–140) as standard in pg/ng protein provided by the manufacturer.

### Histology

#### Sectioning

Hemispheres were cryosectioned sagittally at 10 µm thickness on a Leica CM1950. Sections were mounted on polysine slides (Thermo Scientific) and were stored at −20 °C.

#### Immunofluorescence

Double-and triple immunofluorescence experiments were performed using the following protocol: Wash cryo-sections 2 × 5 min in PBS, block 1 h with normal donkey serum and 0.2% Triton X-100, wash 2 × 2 min in PBS, incubate 1 h in antibody diluent (Dako) with primary antibodies, wash 3 × 5 min, incubate secondary antibodies 30 min in antibody diluents, wash 3 × 5 min in PBS, stain nuclei with DAPI, mount with Mowiol. Detailed information on all primary antibodies used in this study is provided in Table 1.

**Table 1:** List of primary antibodies used for histological evaluations

Species	Antigen	Clone	Source	Item #	Dilution
Rat	Human α-synuclein	15G7	Enzo Life Sciences, Plymouth Meeting, PA	804-258-L001	1:10
Rabbit	Phospho-Ser129 α-synuclein	EP1536Y	Abcam, Cambridge, UK	ab51253	1:2000
Goat	Pan synuclein (human and murine α/β/γ-synuclein)	poly	Santa Cruz Biotechnology, CA	sc-7012	1:200
Mouse	NeuN	A60	EMD Millipore, Temecula, CA	MAB377	1:500
Rabbit	GFAP	poly	Dako	Z0334	1:500
Rat	CD11b	5C6	BioRad Laboratories/AbD Serotec	MCA711	1:1000
Rabbit	IBA1	poly	Proteintech	10904-1-AP	1:200
Mouse	CNPase	11-5B	EMD millipore	MAB326	1:250

Secondary antibodies donkey anti-rat, donkey anti-mouse, donkey anti-rabbit, and donkey anti-goat were labelled with AlexaFluor488, AlexaFluor555, or AlexaFluor 650 fluorophores (Abcam); all secondary antibodies were highly cross-adsorbed to prevent unspecific cross-reactivity. Specificity of secondary antibodies was assessed by omitting primary antibodies on parallel sections. Controls were routinely executed together with regular experiments.

#### Imaging and image analysis

Mosaic images including the entire hippocampus and neocortex were captured using a fully motorized Zeiss AxioImager Z1 microscope equipped with a Zeiss AxioCam MRm camera and Zeiss AxioVision 4.8 software. Images were processed with Image Pro Plus 6.2 (Media Cybernetics).

### Statistics

Group differences were evaluated by unpaired *t*-test, One-way ANOVA or Two-way ANOVA and post hoc tests corrected for multiple comparisons Newman Keuls and Bonferroni’s, respectively. Tests for normal distribution (Kolmogorov–Smirnov test) were performed, and in case of significantly different distributions appropriate non-parametric tests were chosen. GraphPad Prism (v4.03) was used for all calculations and for the preparation of graphs.

## Results

To establish the Line 61 mice as suitable model for in vivo screening of new PD modulating compounds we analyzed these transgenic mice for human and murine α-Syn expression, neuroinflammation, motor deficits and emotional learning deficits over age.

### Human and mouse α-Syn expression levels in Line 61 mice

**Fig. 1:**
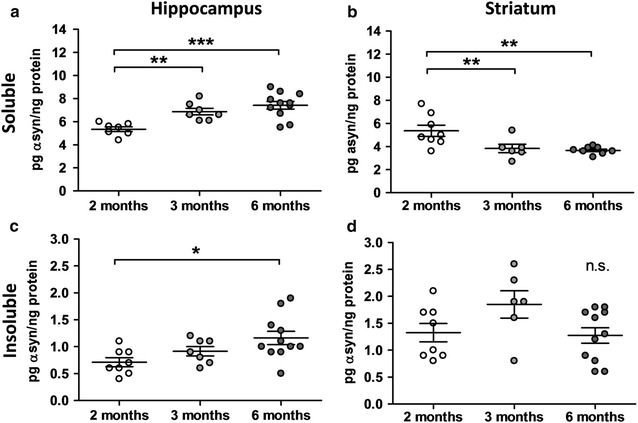
Human α-synuclein protein expression levels in Line 61 mice. Human α-Syn protein expression levels in the soluble (**a**, **b**) and insoluble (**c**, **d**) fraction of the hippocampus (**a**, **c**) and striatum (**b**, **d**) of 2-, 3-, and 6-months old Line 61 mice as measured by MesoScale Discovery. n = 5–11 per group. Mean ± SEM. One-way ANOVA followed by Newman’s Keuls *posthoc* test. *p < 0.05; **p < 0.01; ***p < 0.001

Biochemical evaluation of human α-Syn expression levels in the soluble fraction of the hippocampus of Line 61 mice by MesoScale Discovery platform revealed an increasing expression until 6 months of age (Fig. 1a). By contrast, in the soluble fraction of the striatum the human α-Syn expression levels significantly decreased over age (Fig. 1b). In the insoluble fraction of the hippocampus the human α-Syn expression levels were in general lower compared to the soluble fraction and the increase over age was weaker but still significant (Fig. 1c vs. a). The human α-Syn expression levels in the insoluble fraction of the striatum were also lower compared to the soluble fraction; the levels varied slightly between ages, but differences were not significant (Fig. 1d). In total, the α-Syn expression levels increased in the hippocampus over age while expression in the striatum decreased or rather stayed constant.

**Fig. 2:**
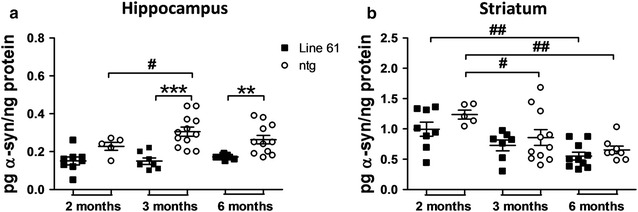
Murine α-synuclein protein expression levels in Line 61 mice. Murine α-Syn protein expression levels in the hippocampus (**a**) and striatum (**b**) of 2-, 3-, and 6-months old Line 61 and ntg mice as measured by MesoScale Discovery. n = 5–12 per group. Mean ± SEM. Two-way ANOVA followed by Bonferroni’s *posthoc* test. *differences between genotypes of the same age, #differences between age groups of one genotype. *p < 0.05; **p < 0.01; ***p < 0.001

Parallel analysis of murine α-Syn levels of 2-, 3- and 6-month old Line 61 mice revealed an increase of murine α-Syn levels in the hippocampus compared to non-transgenic littermates (Fig. 2a), and at the same time a decrease of murine α-Syn in the striatum (Fig. 2b) of Line 61 and also non-transgenic littermates over age. Interestingly, murine α-Syn protein levels of Line 61 mice were in both brain regions slightly lower than in non-transgenic littermates (Fig. 2a, b).

### Morphological alterations

**Fig. 3:**
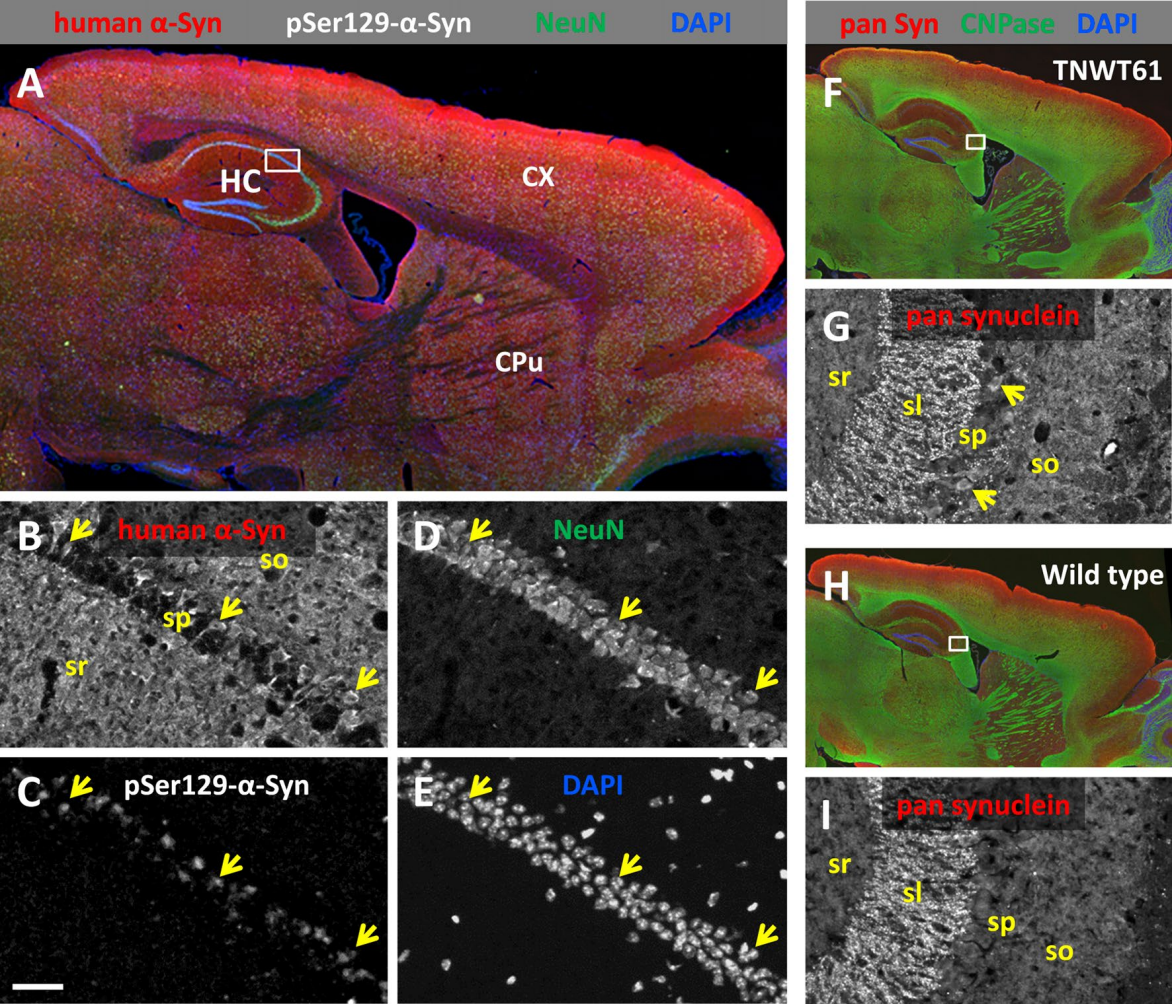
Expression of synuclein in Line 61 mice. **A** A representative section shows expression of transgenic human α-Syn in a male Line 61 mouse at 9 months of age. The transgene is ubiquitously present in gray matter with very high expression levels in the isocortex (red channel). **B**, **C** Magnified single channel images of the hippocampal CA1 region show high human α-Syn levels in a subset of pyramidal cell somata (*arrows*, **B**), and most of these show immunoreactivity for phosphorylated pSer129-α-Syn (**C**). **D**, **E** The overall morphology of the brain is normal, without any obvious occurrence of cell loss in CA1 (NeuN green; DAPI blue). **F**–**I** Immunofluorescent labeling in 7-months old Line 61 mice for total Syn using a pan-specific antibody (α/β/γ-Syn, murine and human), myelinated axons (CNPase green), and DAPI. *CX* cortex, *CPu* corpus putamen, *HC* hippocampus, *sl* stratum lucidum, *so* stratum oriens, *sp* stratum pyramidale, *sr* stratum radiatum. *Scale bar* **A** 500 µm; **F**, **H** 1000 µm; **B**–**E**, **G**, **I** 50 µm

Immunofluorescent labeling of Line 61 brain tissues revealed an overall normal morphology of the brain (Fig. 3A). In the hippocampal CA1 region high human α-Syn levels in a subset of pyramidal cell somata could be observed and most of these show immunoreactivity for phosphorylated pSer129-α-Syn (Fig. 3B, C). No obvious neuronal loss (Fig. 3D, E) or changes in myelination could be detected in 9-month old animals (Fig. 3F–I).

### Neuroinflammation

**Fig. 4:**
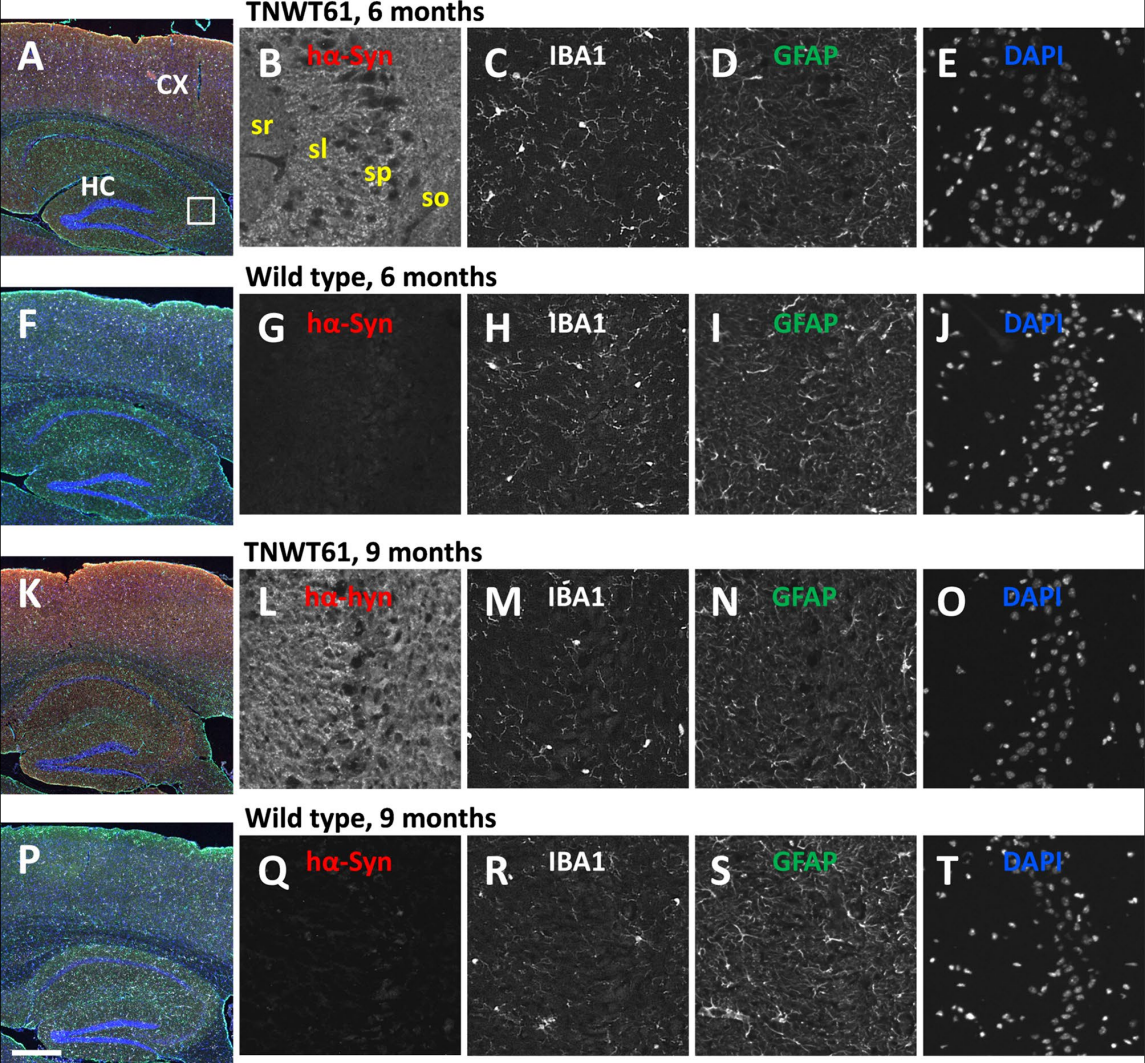
Expression of glia markers in Line 61 and wild type mice. Immunofluorescent labeling patterns of neuroinflammation markers in 6 and 9-months old mice. Magnified single channel images show human α-Syn immunoreactivity (red channel; note absence of signal in wild type mice), IBA1 (microglia white), GFAP (astrocytes green), and DAPI (blue). The general labeling pattern of the gliosis markers is similar in either genotype. *CX* cortex, *HC* hippocampus, *sl* stratum lucidum, *so* stratum oriens, *sp* stratum pyramidale, *sr* stratum radiatum. *Scale bar* **A**, **F**, **K**, **P** 500 µm; grey images: 50 µm

**Fig. 5:**
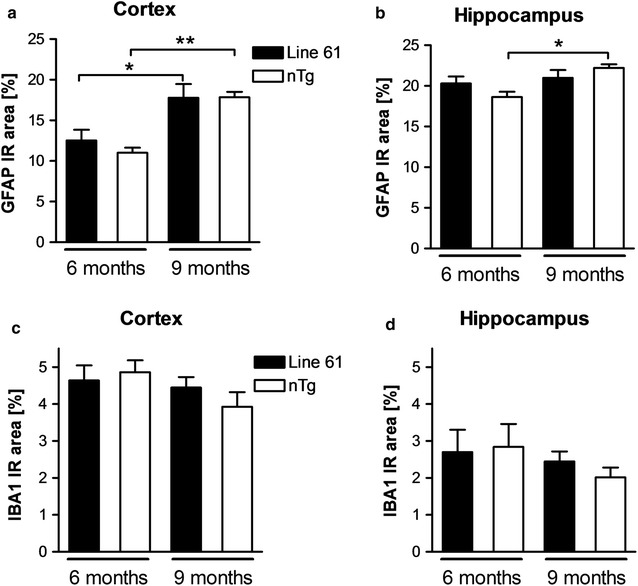
Quantification of neuroinflammation in 6 and 9-months old Line 61 mice. Quantification of astroglia (**a**, **b**) and activated microglia (**c**, **d**) by measurement of GFAP and IBA1 suprathreshold area in percent of area of interest in the cortex (**a**, **c**) and hippocampus (**b**, **d**) of 6 and 9 months old Line 61 mice compared to non-transgenic littermates. n = 6–8 per group. Mean + SEM; One-way ANOVA followed by Bonferroni’s *posthoc* test; *p < 0.05; **p < 0.01

Histological analysis of Line 61 cortical and hippocampal tissue for neuroinflammation revealed a physiological pattern of astrocytes and microglia in the adult brain without any indication of white matter pathology (Fig. 4). Quantification of GFAP and IBA1-immunofluorescence in the cortex and hippocampus of 6 and 9 months old animals revealed no significant alterations in astrogliosis or activated microglia levels in Line 61 mice compared to non-transgenic littermates (Fig. 5a–d). In the cortex and hippocampus an age dependent increase of astrogliosis could be observed in non-transgenic and weaker also in Line 61 mice (Fig. 5a, b).

### Motor performance of Line 61 mice

**Fig. 6:**
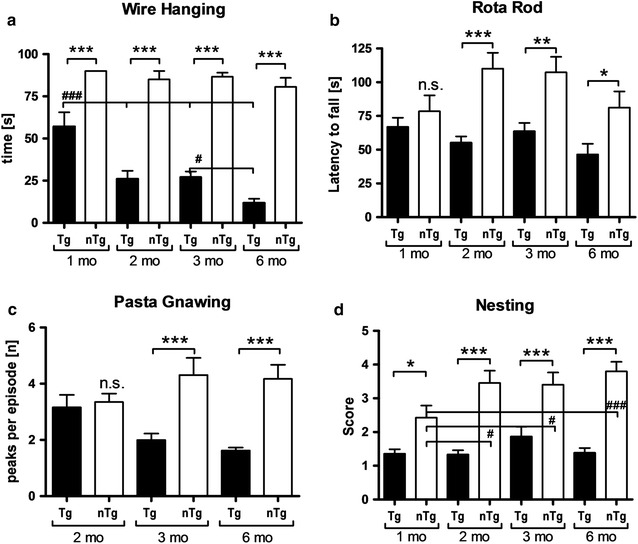
Progressive motor deficits of Line 61 mice measured by four different motor tests. **a** Time in seconds mice hang on the wire in the wire hanging test. **b** Latency to fall from the rotating rod in the RotaRod test. **c** Peaks per gnawing episode in the pasta gnawing test. **d** Scoring of the nest building test (1 bad nest, 5 perfect nest). **a**–**d** n = 10–15 per group; Mean ± SEM. Two-way ANOVA followed by Bonferroni’s *posthoc* test. **b**, **c** 1 outlier excluded. *differences between transgenes. #differences between age groups of one genotype. *p < 0.05; **p < 0.01; ***p < 0.001

**Fig. 7:**
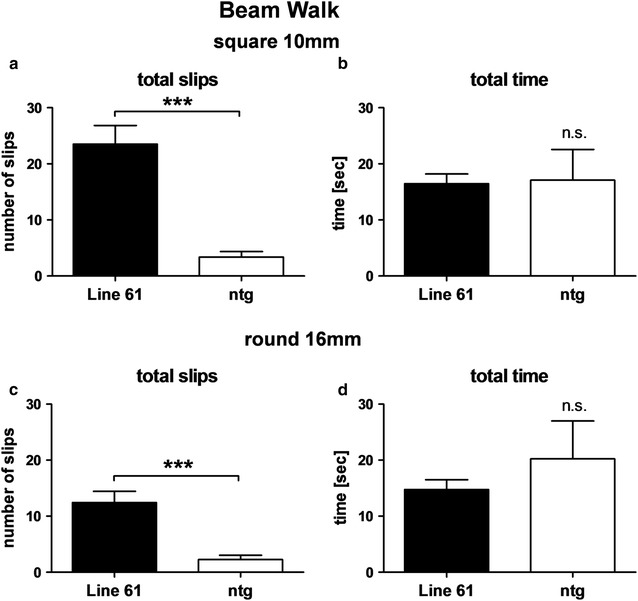
Motor deficits of 6-months old Line 61 mice as measured by Beam walk test. Number of total slips (**a**, **c**) and total time in seconds (**b**, **d**) that Line 61 mice need to traverse the square 10 mm beam (**a**, **b**) or the round 16 mm beam (**c**, **d**) compared to non-transgenic littermates. n = 8 per group. Mean ± SEM, unpaired *t*-test; ***p < 0.001

Already at 1 month of age Line 61 mice exhibited a significantly reduced hanging time in the wire hanging test compared to non-transgenic littermates. These deficits even increased over age while the performance of non-transgenic littermates remained unchanged (Fig. 6a). Additionally, evaluation of Line 61 mice using the RotaRod test revealed first motor deficits as measured by latencies to fall from the rod at an age of 2 months (Fig. 6b). These differences increased from 1 to 2 months of age while deteriorating in adult animals due to an initially better and later poorer performance of non-transgenic littermates (Fig. 6b). To further characterize motor deficits in Line 61 mice, the animal’s biting performance was tested using the pasta gnawing test, which was adapted from [14]. The sound that is produced while eating a dry spaghetti noodle was recorded and the biting peaks per eating episode were measured. The test showed that starting at 3 months of age Line 61 mice eat pasta with a significantly lower number of peaks per episode compared to non-transgenic littermates (Fig. 6c). Neither in Line 61 mice nor in non-transgenic littermates did the number of biting peaks per episode alter over age. In the nest building test first significant differences between genotypes were observed at the age of 1 month. At this age, Line 61 built poorer nests compared to non-transgenic littermates (Fig. 6d). The analysis of nest building over age showed that Line 61 animals built nests of the same low quality, independent of age, while the nest quality of non-transgenic littermates significantly increased over age, thus increasing the differences between the genotypes with increasing age (Fig. 6d). To corroborate the results of these motor tests, 6 months old animals were also tested in the beam walk test by using two different beams. Analyses of the total number of slips in Line 61 mice compared to control animals confirmed the motor deficits observed in younger animals (Fig. 7a, c). The total time animals needed to perform the test did not change in the beam walk test (Fig. 7b, d).

### Learning deficits in Line 61 mice

**Fig. 8:**
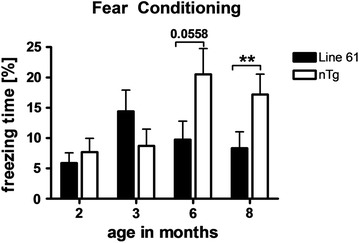
Cognitive deficits of Line 61 mice as measured by the fear conditioning test. Freezing time of 2, 3, 6, and 8-months old Line 61 mice in the fear conditioning test compared to non-transgenic littermates. n = 18 per group. Mean ± SEM, unpaired *t*-test. **p < 0.01. Analysis by two-way ANOVA resulted in no significant differences

Since PD is not only characterized by motor deficits but often also by subcortical dementia, Line 61 mice were tested for emotional learning deficits in the contextual fear conditioning task. Our results show that at an age of 8 months Line 61 mice showed less freezing behavior in the fear conditioning task compared to non-transgenic littermates (Fig. 8) indicating that Line 61 present with learning deficits, a symptom rarely observed in other PD mouse models. Younger animals of 2, 3 and 6 months were also analyzed using the Fear conditioning test, but results did not show any significant differences compared to non-transgenic littermates, although a trend towards disturbed learning behavior could be observed in 6-month old animals (Fig. 8).

## Discussion

The Line 61 mouse model represents the highest expresser of five published founder lines, with a tenfold increased expression of human α-syn compared to human control tissue [8]. The original publication presenting this transgenic mouse model provided encouraging data about the expression profile of the human α-Syn transgene and corresponding α-Syn accumulations in the substantia nigra, laying the cornerstone for the successful introduction of a new and promising mouse model for PD [8]. Here we analyzed human α-Syn protein expression levels over age showing a significant increase in the soluble and insoluble fraction of the hippocampus up to an age of 6 months. The employed Thy-1 promoter is known to be inactive until birth but is rapidly increasing expression to almost 100-fold during early postnatal development [18]. This prompt activation of the promoter can explain why the transgene is already strongly up-regulated in 2-month old Line 61 mice. In the soluble and insoluble fraction of the striatum we observed a significant decrease or rather no altered expression over age of human α-Syn protein expression levels, contrary to values in the hippocampus. These data are surprising given the significant motor deficits in five different motor tests suggesting that the concentration of human α-Syn protein in the striatum is not positively associated with the corresponding motor or cognitive deficit. Additionally, we observed lower murine α-Syn protein levels in Line 61 mice compared to non-transgenic mice, suggesting that over-expression of human α-Syn causes a downregulation of endogenous α-Syn.

Our histological analyses revealed that 6 and 9-months old Line 61 mice present physiological astrogliosis and activated microglia levels in the cortex and hippocampus that slightly increase over age similar to non-transgenic littermates. These data contradict results by others who provide first evidence of neuroinflammation in this mouse model by approximately 9 months [19, 20]. In these publications, different antibodies to label GFAP were used and the analyzed cortical regions might vary, so differences could be caused by systematic differences and it cannot be excluded that analysis of even older animals would reveal an increase in neuroinflammatory marker. Animals used for the here presented study are genetically separated from the original mouse model [8] for already more than 10 years, thus genetic differences between the analyzed mice might also have caused the observed difference in neuroinflammation. In PD patients, neuroinflammation is a common neuropathological feature that is discussed to be induced by aggregeted α-Syn but also be able to modulate α-Syn processing and clearance [21]. Recent research suggests that mutated α-Syn promotes microglia activation much stronger than wildtype α-Syn [22]. The physiological neuroinflammation levels in Line 61 mice up to an age of 9 months suggest that induction of neuroinflammation by wildtype human α-Syn is not a prerequisite and neuroinflammatory events in PD might thus not fully depend on α-Syn.

Previous reports about Line 61 mice provided first proof of motor deficits in Line 61 mice as early as 2 months of age [11, 12, 23]. Due to the early onset of motor deficits, the Line 61 mice were already used to study the effect of different compounds, like paraquat and dopamine agonists on α-Syn [23, 24]. Here we analyzed the Line 61 mice for motor deficits in a whole series of motor tests and were able to validate previous results of other laboratories; in addition we demonstrate first motor deficits in 1-month old Line 61 animals that worsen over age [11, 12, 23, 24]. The differences between Line 61 and non-transgenic littermates in the wire hanging test are already significant at an age of 1 month and thus could be used to distinguish transgenic from non-transgenic mice. This test provides strong evidence for the pathological impact of the human α-Syn protein, since the expression, regulated by the Thy-1 promoter, starts only after birth [12] and still causes severe motor changes already at the age of 1 month. Classically, the nest building test assesses maternal or social behavior. However, to perform the test properly, motor abilities like orofacial and forelimb movements are essential [25], activities that are all dopamine-dependent [26] and already shown to be dysfunctional in the striatum of Line 61 mice [27–30]. The nest building test can therefore also be considered as a test for motor skills, supporting results from basic motor tests like the bin cotton use [11] and the pasta gnawing test in our study. In combination with the results of the bin cotton test it is likely that the observed nest building deficits are exclusively caused by motor shortfalls since the analysis over age shows that nest building improves only in older non-transgenic mice. Whether Line 61 mice also present maternal or social discrepancies remains unclear. The pasta gnawing test analyzes the gnawing speed and thus a very basic motor skill relevant for the primary need of food manipulation [15]. First significant differences could be observed at the age of 3 months, hence 2 months later as in the wire hanging test. These results suggest that the changes observed with the diverse tests depend on different brain regions that are differently affected by the α-Syn pathology.

The results of the emotional learning test suggest that Line 61 mice present amygdala dependent emotional learning deficits as proven with the contextual fear conditioning test and therefore portray similar deficits as observed in patients with subcortical dementia but without cortical pathology. Since PD in humans is not only characterized by motor deficits like rigor, bradykinesia, tremor and postural instability but also by non-motor symptoms like hyposmia, gastrointestinal dysfunction, anxiety and cognitive decline [31] proper animal models for PD should also present as many of these non-motor deficits as possible [32]. It has been previously observed that Line 61 mice show olfactory impairments at an age of 5–6 months [33] as well as altered basal and stress-induced propulsive colonic motility at an age of 12 months [34]. Together with the data in the present study on motor and emotional learning deficits of Line 61 mice, this transgenic mouse model is not only excellently applicable for the analysis of typical motor deficits but also for non-motor discrepancies that are typical for PD.

## Conclusion

In summary, our data show for the first time progressive motor deficits of Line 61 mice that can already be observed at an age of 1 month. The results are in agreement with earlier results of Line 61 mice and emphasize the use of this model to test new compounds against PD and other synucleinopathies.

## Abbreviations

AAALAC: Association for Assessment and Accreditation of Laboratory Animal Care
ntg: non-transgenic littermates
PD: Parkinson’s disease
RT: room temperature
α-Syn: α-synuclein
tg: transgenic

## References

[1] Spillantini MG, Schmidt ML, Lee VMY, Trojanowski JQ, Jakes R, Goedert M (1997). Alpha-synuclein in Lewy bodies. Nature.

[2] Polymeropoulos MH, Lavedan C, Leroy E, Ide SE, Dehejia A, Dutra A, Pike B, Root H, Rubenstein J, Boyer R (1997). Mutation in the alpha-synuclein gene identified in families with Parkinson’s disease 7. Science.

[3] Kruger R, Kuhn W, Muller T, Woitalla D, Graeber M, Kosel S, Przuntek H, Epplen JT, Schols L, Riess O (1998). Ala30Pro mutation in the gene encoding alpha-synuclein in Parkinson’s disease. Nat Genet.

[4] Ibánez P, Bonnet AM, Debarges B, Lohmann E, Tison F, Pollak P, Agid Y, Dürr A, Brice A (2004). Causal relation between α-synuclein gene duplication and familial Parkinson’s disease. Lancet.

[5] Chartier-Harlin MC, Kachergus J, Roumier C, Mouroux V, Douay X, Lincoln S, Levecque C, Larvor L, Andrieux J, Hulihan M (2004). α-Synuclein locus duplication as a cause of familial Parkinson’s disease. Lancet.

[6] Singleton AB, Farrer M, Johnson J, Singleton A, Hague S, Kachergus J, Hulihan M, Peuralinna T, Dutra A, Nussbaum R (2003). Alpha-synuclein locus triplication causes Parkinson’s disease. Science.

[7] Masliah E, Rockenstein E, Veinbergs I, Mallory M, Hashimoto M, Takeda A, Sagara Y, Sisk A, Mucke L (2000). Dopaminergic loss and inclusion body formation in alpha-synuclein mice: implications for neurodegenerative disorders. Science.

[8] Rockenstein E, Mallory M, Hashimoto M, Song D, Shults CW, Lang I, Masliah E (2002). Differential neuropathological alterations in transgenic mice expressing alpha-synuclein from the platelet-derived growth factor and Thy-1 promoters. J Neurosci Res.

[9] Hashimoto M, Rockenstein E, Masliah E (2003). Transgenic models of alpha-synuclein pathology: past, present, and future. Ann N Y Acad Sci.

[10] Shults CW, Rockenstein E, Crews L, Adame A, Mante M, Larrea G, Hashimoto M, Song D, Iwatsubo T, Tsuboi K (2005). Neurological and neurodegenerative alterations in a transgenic mouse model expressing human alpha-synuclein under oligodendrocyte promoter: implications for multiple system atrophy. J Neurosci.

[11] Fleming SM, Salcedo J, Fernagut PO, Rockenstein E, Masliah E, Levine MS, Chesselet MF (2004). Early and progressive sensorimotor anomalies in mice overexpressing wild-type human alpha-synuclein. J Neurosci.

[12] Chesselet MF, Richter F, Zhu C, Magen I, Watson MB, Subramaniam SR (2012). A progressive mouse model of Parkinson’s disease: the Thy1-aSyn (“Line 61”) mice. Neurotherapeutics.

[13] Dunham NW, Miya TS (1957). A note on a simple apparatus for detecting neurological deficit in rats and mice. J Am Pharm Assoc Am Pharm Assoc.

[14] Kane JR, Ciucci MR, Jacobs AN, Tews N, Russell JA, Ahrens AM, Ma ST, Britt JM, Cormack LK, Schallert T (2011). Assessing the role of dopamine in limb and cranial-oromotor control in a rat model of Parkinson’s disease. J Commun Disord.

[15] Rabl R, Horvath A, Breitschaedel C, Flunkert S, Roemer H, Hutter-Paier B (2016). Quantitative evaluation of orofacial motor function in mice: the pasta gnawing test, a voluntary and stress-free behavior test. J Neurosci Methods.

[16] Deacon RM (2006). Assessing nest building in mice. Nat Protoc.

[17] Wills J, Credle J, Haggerty T, Lee JH, Oaks AW, Sidhu A (2011). Tauopathic changes in the striatum of A53T alpha-synuclein mutant mouse model of Parkinson’s disease. PLoS ONE.

[18] Morris R (1985). Thy-1 in developing nervous tissue. Dev Neurosci.

[19] Rockenstein E, Nuber S, Overk CR, Ubhi K, Mante M, Patrick C, Adame A, Trejo-Morales M, Gerez J, Picotti P (2014). Accumulation of oligomer-prone alpha-synuclein exacerbates synaptic and neuronal degeneration in vivo. Brain.

[20] Valera E, Mante M, Anderson S, Rockenstein E, Masliah E (2015). Lenalidomide reduces microglial activation and behavioral deficits in a transgenic model of Parkinson’s disease. J Neuroinflammation.

[21] Sanchez-Guajardo V, Barnum CJ, Tansey MG, Romero-Ramos M (2013). Neuroimmunological processes in Parkinson’s disease and their relation to alpha-synuclein: microglia as the referee between neuronal processes and peripheral immunity. ASN Neuro.

[22] Hoenen C, Gustin A, Birck C, Kirchmeyer M, Beaume N, Felten P, Grandbarbe L, Heuschling P, Heurtaux T (2016). Alpha-synuclein proteins promote pro-inflammatory cascades in microglia: stronger effects of the A53T mutant. PLoS ONE.

[23] Fleming SM, Salcedo J, Hutson CB, Rockenstein E, Masliah E, Levine MS, Chesselet MF (2006). Behavioral effects of dopaminergic agonists in transgenic mice overexpressing human wildtype α-synuclein. Neuroscience.

[24] Fernagut PO, Hutson CB, Fleming SM, Tetreaut NA, Salcedo J, Masliah E, Chesselet MF (2007). Behavioral and histopathological consequences of paraquat intoxication in mice: effects of alpha-synuclein over-expression. Synapse.

[25] Sager TN, Kirchhoff J, Mork A, Van Beek J, Thirstrup K, Didriksen M, Lauridsen JB (2010). Nest building performance following MPTP toxicity in mice. Behav Brain Res.

[26] Silva MR, Bernardi MM, Felicio LF (2001). Effects of dopamine receptor antagonists on ongoing maternal behavior in rats. Pharmacol Biochem Behav.

[27] Wu N, Joshi PR, Cepeda C, Masliah E, Levine MS (2010). Alpha-synuclein overexpression in mice alters synaptic communication in the corticostriatal pathway. J Neurosci Res.

[28] Clark J, Clore EL, Zheng K, Adame A, Masliah E, Simon DK (2010). Oral N-acetyl-cysteine attenuates loss of dopaminergic terminals in alpha-synuclein overexpressing mice. PLoS ONE.

[29] Lam HA, Wu N, Cely I, Kelly RL, Hean S, Richter F, Magen I, Cepeda C, Ackerson LC, Walwyn W (2011). Elevated tonic extracellular dopamine concentration and altered dopamine modulation of synaptic activity precede dopamine loss in the striatum of mice overexpressing human alpha-synuclein. J Neurosci Res.

[30] Cabeza-Arvelaiz Y, Fleming SM, Richter F, Masliah E, Chesselet MF, Schiestl RH (2011). Analysis of striatal transcriptome in mice overexpressing human wild-type alpha-synuclein supports synaptic dysfunction and suggests mechanisms of neuroprotection for striatal neurons. Mol Neurodegener.

[31] Chaudhuri KR, Healy DG, Schapira AH (2006). Non-motor symptoms of Parkinson’s disease: diagnosis and management. Lancet Neurol.

[32] Taylor TN, Greene JG, Miller GW (2010). Behavioral phenotyping of mouse models of Parkinson’s disease. Behav Brain Res.

[33] Fleming SM, Tetreault NA, Mulligan CK, Hutson CB, Masliah E, Chesselet MF (2008). Olfactory deficits in mice overexpressing human wildtype alpha-synuclein. Eur J Neurosci.

[34] Wang L, Fleming SM, Chesselet MF, Tache Y (2008). Abnormal colonic motility in mice overexpressing human wild-type alpha-synuclein. NeuroReport.

